# Вовлеченность эссенциальных микроэлементов в патогенез заболеваний щитовидной железы: диагностические маркеры и аналитические методы определения

**DOI:** 10.14341/probl13402

**Published:** 2024-04-02

**Authors:** Е. А. Трошина, Н. М. Платонова, Е. С. Сенюшкина, В. А. Иоутси, Е. С. Смолин, Л. В. Никанкина, З. Т. Зураева

**Affiliations:** Национальный медицинский исследовательский центр эндокринологии; Национальный медицинский исследовательский центр эндокринологии; Национальный медицинский исследовательский центр эндокринологии; Национальный медицинский исследовательский центр эндокринологии; Национальный медицинский исследовательский центр эндокринологии; Национальный медицинский исследовательский центр эндокринологии; Национальный медицинский исследовательский центр эндокринологии

**Keywords:** микроэлементы, йод, цинк, селен, щитовидная железа, йододефицитные заболевания, узловой зоб, аутоиммунные заболевания щитовидной железы, масс-спектрометрия

## Abstract

**ЦЕЛЬ:**

ЦЕЛЬ. Изучить роль йода, селена и цинка в патогенезе йододефицитных и аутоиммунных заболеваний щитовидной железы (ЩЖ) и научно обосновать выбор биомаркеров обеспеченности и аналитических методов определения.

**МАТЕРИАЛЫ И МЕТОДЫ:**

МАТЕРИАЛЫ И МЕТОДЫ. С помощью тандемной масс-спектрометрии с ионизацией в индуктивно связанной плазме (Agilent 8900 ICP-MS Triple Quad) измерена концентрация йода (I), селена (Se) и цинка (Zn) в сыворотке крови; методом хемилюминесцентного иммунноанализа на автоматическом анализаторе Architect i2000 — ТТГ и АТ-ТПО в сыворотке крови; методом иммуноферментного анализа — ZnT8A; биохимическим методом — ЩФ, SOD1 у 1150 человек в возрасте от 18 до 65 лет (средний возраст обследуемых составил 40±5 лет). Ультразвуковое исследование (УЗИ) ЩЖ выполнено в положении лежа с использованием портативного ультразвукового аппарата LOGIQe с мультичастотным линейным датчиком 10–15 МГц, в ходе исследования оценивали объем ЩЖ, наличие узловых образований и их характеристики по классификации TIRADS, структуру ЩЖ и ее эхогенность.

**РЕЗУЛЬТАТЫ:**

РЕЗУЛЬТАТЫ. В нашем исследовании медианная концентрация йода в сыворотке крови составила 60,68 мкг/л (n=1150) без существенной разницы между полами. У 2% выявлен уровень йода в сыворотке крови менее 30 мкг/л. В части полученных образцов (n=57) у 19% было выявлено пониженное содержание йода в липофильной фракции — менее 10% от общего. В этих образцах были проведены дополнительные исследования ТТГ, общих и свободных фракций Т3 и Т4. В результате все показатели укладывались в пределы нормальных значений, что свидетельствует об отсутствии влияния на функцию ЩЖ снижения содержания йода в липофильной фракции. При сравнительном анализе ранее полученных нами результатов определения йода в моче церий-арсенитным методом и методом масс-спектрометрии с индуктивно связанной плазмой установлено, что оба метода в целом сопоставимы. Медианная концентрация селена составила 83,38 мкг/л, что соответствует референсным значениям. Доля лиц с уровнем селена в сыворотке крови менее 40 мкг/л составила 2,2%. Проведен сравнительный анализ групп пациентов с концентрацией селена в сыворотке крови менее 100 мкг/л и более 100 мкг/л; в группе с низконормальными показателями селена частота встречаемости аутоиммунной патологии ЩЖ на 5% выше, чем в группе сравнения. 60,3% взрослого населения имели уровень цинка менее 1000 мкг/л. Медианная концентрация цинка в сыворотке крови составила 632,9 мкг/л. В регионах с дефицитом цинка частота встречаемости аутоиммунных заболеваний (АИЗ) ЩЖ и узлового/многоузлового зоба в среднем на 10% выше, чем в регионах с оптимальной обеспеченностью цинком. Не выявлено взаимосвязи между содержанием в сыворотке крови цинка и антител к транспортеру цинка (ZnT8A), щелочной фосфатазы (ЩФ) и супероксиддисмутазы (SOD1), в т.ч. в сопоставлении полученных данных у носителей АТ-ТПО и в группе сравнения (среди носителей АТ-ТПО: медианная концентрация цинка составила 644,4 мкг/л, SOD1 — 117,2 нг/мл, ЩФ — 70,3 Ед/л, антитела к ZnT8A — 249,8; в группе сравнения — медианная концентрация цинка — 744,6 мкг/л, SOD1 — 102,4 нг/мл, ЩФ — 66,1 Ед/л, антитела к ZnT8A — 242).

Таким образом, на основании полученных данных подтверждена взаимосвязь между тиреоидной патологией и микронутриентными дефицитами. Не получено убедительных доказательств по исследованию дополнительных диагностических маркеров дефицита Zn, что ставит под сомнение целесообразность их определения в рутинной практике. Метод ИСП-МС позволил предложить собственные референсные значения I, Se, Zn и сопоставим с церий-арсенитным методом по чувствительности и специфичности при исследовании I в моче. Однако, ввиду технических особенностей и ограничения по объему выборки изучаемой популяции, требует дальнейшего совершенствования.

## Abstract (en)

**AIM:**

AIM. To study the role of iodine, selenium and zinc in the pathogenesis of iodine deficiency and autoimmune thyroid diseases and scientifically substantiate the choice of security biomarkers and analytical methods for determination.

**MATERIALS AND METHODS:**

MATERIALS AND METHODS. Serum iodine (I), selenium (Se), and zinc (Zn) concentrations were measured using inductively coupled plasma ionization tandem mass spectrometry (Agilent 8900 ICP-MS Triple Quad); by chemiluminescent immunoassay on an automatic analyzer Architect i2000 — TSH and AT-TPO in blood serum; by enzyme immunoassay — ZnT8A; biochemical method — ALP, SOD1 in 1150 people aged from 18 to 65 years (the average age of the subjects was 40±5 years). Ultrasound of the thyroid gland was performed in the supine position using a portable ultrasound machine LOGIQe with a multifrequency linear sensor 10–15 MHz; during the study, the volume of the thyroid gland, the presence of nodules and their characteristics according to the TIRADS classification, the structure of the thyroid gland and its echogenicity were assessed.

**RESULTS:**

RESULTS. In our study, the median serum iodine concentration was 60.68 μg/L (n=1150), with no significant difference between sexes. In 2%, the level of iodine in the blood serum was less than 30 mcg/l. Among the samples obtained (n=57), 19% were found to have a reduced iodine content in the lipophilic fraction — less than 10% of the total. In these samples, additional studies were performed on TSH, total and free T3 and T4 fractions. As a result, all indicators fell within the normal range, which indicates that there was no effect on thyroid function from a decrease in iodine content in the lipophilic fraction. In a comparative analysis of our previously obtained results of determining iodine in urine using the cerium-arsenite method and the inductively coupled plasma mass spectrometry method, it was found that both methods are generally comparable. The median selenium concentration was 83.38 µg/l, which corresponds to the reference values. The proportion of individuals with serum selenium levels less than 40 μg/L was 2.2%. A comparative analysis of groups of patients with serum selenium concentrations of less than 100 µg/l and more than 100 µg/l was carried out; in the group with low-normal selenium levels, the incidence of autoimmune thyroid pathology is 5% higher than in the comparison group. 60.3% of the adult population had zinc levels less than 1000 μg/L. The median serum zinc concentration was 632.9 μg/L. In regions with zinc deficiency, the incidence of autoimmune diseases (AI) of the thyroid gland and nodular / multinodular goiter is on average 10% higher than in regions with optimal zinc supply. There was no relationship between the content of zinc in the blood serum and antibodies to the zinc transporter (ZnT8A), alkaline phosphatase (ALP) and superoxide dismutase (SOD1), including in comparison of the data obtained in carriers of AT-TPO and in the comparison group (among carriers of AT-TPO: the median concentration of zinc was 644.4 μg/l, SOD1 — 117.2 ng/ml, ALP — 70.3 U/l, antibodies to ZnT8A — 249.8; in the comparison group — median zinc concentration — 744.6 μg/l, SOD1 — 102.4 ng/ml, ALP — 66.1 U/l, antibodies to ZnT8A — 242).

Thus, based on the data obtained, the relationship between thyroid pathology and micronutrient deficiencies was confirmed. No convincing evidence has been obtained on the study of additional diagnostic markers of Zn deficiency, which casts doubt on the advisability of their determination in routine practice. The ICP-MS method made it possible to propose its own reference values for I, Se, Zn and is comparable to the cerium-arsenite method in sensitivity and specificity when studying I in urine. However, due to technical features and limitations in sample size, the study population requires further improvement.

## ОБОСНОВАНИЕ

Актуальность изучения фундаментальных возможных общих механизмов, лежащих в основе йододефицитных (ЙДЗ) и аутоиммунных заболеваний щитовидной железы (АИЗ ЩЖ), ассоциированных с различной обеспеченностью эссенциальными микроэлементами, очевидна. Взаимосвязь между тиреоидной патологией и нутриентными дефицитами изучают уже длительное время. За много лет предложены различные методы определения микроэлементов в различных биологических средах. Выбор оптимального метода является крайне важным и позволяет лучше понять механизмы развития и оценить прогноз йододефицитных и аутоиммунных тиреопатий как у конкретного человека, так и в популяции в целом [1–4].

Крупномасштабные исследования свидетельствуют о том, что ситуация с обеспеченностью населения разных стран различными витаминами и микроэлементами достаточно неоднозначная. Среди здоровых женщин репродуктивного возраста только 5% обеспечены всеми витаминами и микроэлементами. В мире около 1/3 населения испытывают дефицит одного или нескольких микронутриентов, чаще всего йода (I), железа (Fe), цинка (Zn), витамина А и фолиевой кислоты. В России, как и в странах Западной Европы, ситуация с реальной обеспеченностью населения микронутриентами практически одинакова. По результатам многочисленных исследований выявлены множественные ассоциации между микронутриентными дефицитами и различными коморбидными патологиями, включающими заболевания щитовидной железы (ЩЖ) [2][4][5].

Ключевая роль в синтезе и метаболизме тиреоидных гормонов принадлежит йоду (I), селену (Se) и цинку (Zn). В исследованиях последних лет приводятся и данные об их участии в процессах аутоиммунитета. Не исключено, что дефицит этих микроэлементов приводит не только к формированию диффузного и узлового зоба и развитию гипотиреоза, но является триггером синтеза и(или) действия антитиреоидных аутоантител. Изучение роли сочетанного дефицита микроэлементов в развитии заболеваний ЩЖ представляется актуальной проблемой. Однако ни один из описанных феноменов не имеет четких научных доказательств, возможно, по причине существенных различий в методах определения эссенциальных микроэлементов, приводящих к несопоставимым между собой результатам.

Йод — важнейший микроэлемент, необходимый для синтеза гормонов щитовидной железы, которые регулируют метаболические процессы в большинстве клеток и играют ведущую роль в росте и развитии организма человека. ЙДЗ являются глобальной проблемой для многих стран мира, целесообразность их профилактики прежде всего связана с предотвращением нарушения формирования головного мозга на этапе эмбрионального развития и снижением заболеваемости тиреопатиями в любом возрасте. Экскреция I с мочой — основной эпидемиологический показатель, характеризующий обеспеченность I населения; является главным критерием оценки тяжести йодного дефицита [6]. Суточная потребность человека в I составляет 150 мкг для взрослых и подростков, 250 мкг для беременных и кормящих женщин [6–8].

Селен — один из важных и наиболее изучаемых в настоящее время микроэлементов. Выполняет свои биохимические функции в составе селенсодержащих белков. К основным белкам, локализующимся в больших количествах в ЩЖ, относятся ферменты — глутатионпероксидаза (GPX), дейодиназа (ID), тиоредоксинредуктаза (TXNRD), селенопротеины (SELENOP, SELENON, SELENOS). Они участвуют во многих разнообразных биологических процессах, включая синтез ДНК, антиоксидантную защиту, метаболизм гормонов ЩЖ, иммунные реакции и т.д.

Дефицит Se снижает способность Т4 превращаться в Т3. Недостаточное потребление данного микроэлемента с пищей влияет на развитие различной патологии ЩЖ. Для пациентов с АИЗ ЩЖ в дебюте заболевания характерны низкие концентрации Se в сыворотке крови. Дефицит Se инициирует и способствует прогрессированию болезни Грейвса и хронического аутоиммунного тиреоидита (ХАИТ), увеличивает риск развития узлового зоба, эндокринной офтальмопатии и рака щитовидной железы. Адекватное потребление Se способствует более быстрому достижению эутиреоза как при болезни Грейвса, так и при гипотиреозе в исходе ХАИТ. Нормы физиологической потребности в Se составляют: для мужчин 70 мкг/сут, для женщин — 55 мкг/сут. Биомаркерами обеспеченности Se является его уровень в сыворотке крови и активность GPX в эритроцитах [2][9][10].

Цинк — микроэлемент, участвующий во многих процессах, важнейших для эндокринной системы: синтезе и секреции инсулина, регуляции синтеза и действии гормонов ЩЖ, регуляции артериального давления. Данный микроэлемент необходим для правильного функционирования ID первого типа, для связывания T3 с рецепторами тиреоидных гормонов и играет ключевую роль во взаимодействии рецепторов тиреоидных гормонов с генами-мишенями. Zn участвует в превращении Т4 в метаболически активный Т3. Дефицит Zn подавляет синтез гормонов ЩЖ и нарушает связывание Т3 с ядерными рецепторами, что приводит к гипотиреозу. Дефицит Zn может снизить и эффективность препаратов I, назначаемых как для профилактики, так и для лечения ЙДЗ. Наряду с дефицитом других микроэлементов из-за низкого уровня Zn существенно повышается риск развития диффузного и узлового зоба. Дефицит Zn также способствует увеличению выработки антител к ткани ЩЖ, может лежать в основе аутоиммунных тиреопатий. Восполнение дефицита Zn замедляет процессы разрушения клеток ЩЖ. Физиологический уровень поступления Zn для взрослого здорового человека составляет 12 мг/сут. Биомаркером обеспеченности Zn является уровень Zn в сыворотке крови и в суточной моче. Согласно данным многих исследований, в отношении Zn важно также учитывать дополнительные маркеры обеспеченности, такие как активность SOD1, ЩФ и др. [2][3][11].

Концентрация того или иного микроэлемента в биологических средах не всегда отражает его точное содержание в организме и требует поиска дополнительных специфических индикаторов. Экскреция I с мочой на сегодняшний день является основным эпидемиологическим показателем, характеризующим обеспеченность I населения; является главным критерием оценки тяжести йодного дефицита. Определение I в крови в настоящее время производится в виде общего показателя, то есть содержание I в образце независимо от формы его связывания. В настоящее время, как в России, так и за рубежом, практически не существует данных по определению I в разных фракциях сыворотки крови с целью определения влияния йодного дефицита на отдельные ее компоненты. Согласно некоторым исследованиям, для оценки обеспеченности Zn и Se является важным не только их определение в сыворотке крови и моче, но и анализ таких показателей, как ЩФ, SOD1, GPX и др. в сыворотке крови как дополнительных специфических индикаторов. Дефицит Zn и Se приводит к снижению активности SOD1, ЩФ и GPX.

SOD1 — антиоксидантный фермент, состоящий из двух субъединиц, каждая из которых имеет активный центр из атомов Zn и меди (Cu). SOD1 имеет нестабильную структуру. Стабильность структуры обеспечивается за счет атома Zn, а атом Cu действует как каталитический центр. SOD1 катализирует распад супероксидного аниона на молекулу О2 и Н2О2. Нарушение метаболизма Zn отрицательно влияет на синтез и функцию системы антиоксидантной защиты, что приводит к окисидативному стрессу. При дефиците Zn снижается активность SOD1 [11].

ЩФ — изофермент, катализирующий гидролиз органических эфиров фосфорной кислоты, присутствующих во внеклеточном пространстве. Zn является одним из важных кофакторов, определяющих биологическую активность данного фермента. Активность ЩФ снижается при дефиците Zn [12].

GPX — основной селенсодержащий фермент, предотвращающий накопление в тканях свободных радикалов и обеспечивающий антиоксидантную защиту. В ЩЖ GPX присутствует в больших количествах. Экспрессия GPХ 1 типа наиболее чувствительна к концентрации Se и снижается при низких его уровнях [9][13].

ZnT8 — подтип транспортера Zn, способствующий транспорту Zn из клетки в субклеточные компартменты. Экспрессируется в фолликулярных и С-клетках ЩЖ у пациентов с болезнью Грейвса и нетоксическим узловым зобом. По данным некоторых исследований, у пациентов с болезнью Грейвса выявляется высокая концентрация аутоантител к транспортеру цинка — ZnT8A. Восполнение дефицита Zn, как при гипотиреозе, так и при тиреотоксикозе, способствует менее выраженному разрушению клеток ЩЖ [5][14].

Для оценки биологических взаимовлияний, ведущих к нарушениям тиреоидной функции, требуются исследования с применением сверхточных химических методов измерения макро- и микроэлементов. Научное обоснование оптимального метода определения микроэлементов позволит разработать методику оценки обеспеченности эссенциальными микроэлементами, применимую как для эпидемиологических, так и для клинических исследований. В настоящее время, как в России, так и за рубежом, используются различные аналитические методы определения йода в моче и сыворотке крови, за последние несколько лет чаще — масс-спектрометрия с индуктивно связанной плазмой (ИСП-МС). ИСП-МС является золотым стандартом определения микроэлементов в различных биологических субстратах. Преимуществами данного метода перед другими являются многоэлементность определения в сочетании с высокой чувствительностью. Для более чем 90% микроэлементов пределы обнаружения (LoD) ниже 1 мкг/л в водном растворе [1][15–17].

## ЦЕЛЬ ИССЛЕДОВАНИЯ

Изучить роль йода, селена и цинка в патогенезе йододефицитных и аутоиммунных заболеваний щитовидной железы и научно обосновать выбор биомаркеров обеспеченности и аналитических методов определения.

## МАТЕРИАЛЫ И МЕТОДЫ

## Место и время проведения исследования

Место проведения

Исследование проведено одномоментно в рамках выездных мероприятий ГНЦ РФ ФГБУ «НМИЦ эндокринологии» Минздрава России в пяти регионах РФ: Респ. Крым (09.2020 г.), Респ. Тыва (10.2020 г.), Брянская обл. (05.2021 г.), Тульская обл. (05.2022 г.), Чеченская Респ. (06.2022 г.). Протокол исследования одобрен на заседании этического комитета ФГБУ «НМИЦ эндокринологии» Минздрава России от 25.03.2020 (протокол № 5).

Йодная обеспеченность жителей всех вышеперечисленных регионов была одинаковой и соответствовала дефициту йода легкой и средней степени тяжести [18][19].

## Изучаемые популяции (одна или несколько)

1150 человек — мужчины и женщины в возрасте от 18 до 65 лет (средний возраст обследуемых составил 40±5 лет).

Критерии включения: пол: мужчины и женщины; возраст: 18 лет и старше; подписание участником исследования информированного согласия на участие в исследовании.

Критерии исключения: период беременности и лактации; наличие любых острых либо декомпенсация хронических заболеваний на момент включения в исследование; отказ пациента от участия в исследовании.

## Способ формирования выборки из изучаемой популяции (или нескольких выборок из нескольких изучаемых популяций)

Сплошной способ формирования выборки.

## Дизайн исследования

Одномоментное сравнительное исследование.

## Методы

У обследуемых проведен сбор анамнеза, осмотр врача-эндокринолога, пальпация ЩЖ, забор крови для оценки уровня ТТГ, АТ-ТПО, I, Se, Zn, SOD1, ZnT8A; выполнено ультразвуковое исследование щитовидной железы (УЗИ ЩЖ).

Уровни I, Se и Zn в сыворотке крови оценены методом ИСП-МС. Анализ проведен на масс-спектрометре Agilent 8900 ICP-MS Triple Quad. В части образцов сыворотки крови (n=57) уровень йода с предварительно экстрагированными из нее производными тироксина и трийодтиронина оценен методом ИСП-МС.

Уровни ТТГ и АТ-ТПО в сыворотке крови оценены методом хемилюминесцентного иммуноанализа на автоматическом анализаторе Architect i2000 (Abbott).

Уровни SOD1 и ZnT8A определены с помощью коммерческих иммуноферментных наборов:

-антитела к транспортеру цинка (ZnT8A) (BIOVENDOR LABORATORY MEDICINE, INC., Чешская республика);

-супероксиддисмутаза 1 (Cu/ZnSOD) (BENDER MEDSYSTEMS GMBH, Австрия).

УЗИ ЩЖ проведено в соответствии с рекомендациями ВОЗ с использованием портативного ультразвукового аппарата LOGIQe (China) с мультичастотным линейным датчиком 10–15 МГц в положении лежа. Объем ЩЖ будет рассчитываться по формуле:

Vщж = [(Шпр. × Дпр. × Тпр.) + (Шл. × Дл. × Тл.)] × 0,479,

где Vщж — объем щитовидной железы;

Шпр. — ширина правой доли щитовидной железы;

Шл. — ширина левой доли щитовидной железы;

Дпр. — длина правой доли щитовидной железы;

Дл. — длина левой доли щитовидной железы;

Тпр. — толщина правой доли щитовидной железы;

Тл. — толщина левой доли щитовидной железы.

## Статистический анализ

Статистический анализ проведен с помощью программ Excel 2013 (Microsoft, USA), Statistica v.12 (Statsoft, USA).

## Этическая экспертиза

Протокол исследования одобрен на заседании этического комитета ФГБУ «НМИЦ эндокринологии» Минздрава России от 25 марта 2020 г. (протокол № 5).

## РЕЗУЛЬТАТЫ

## Определение уровня йода в сыворотке крови

Медианная концентрация I в сыворотке крови составила 60,68 мкг/л (n=1150) без существенной разницы между полами, что соответствует целевым значениям (рис. 1, рис. 2, табл. 1). У 2% (n=23) выявлен уровень I в сыворотке крови менее 30 мкг/л (рис. 1). В части полученных образцов (n=57) у 19% было выявлено пониженное содержание I в липофильной фракции — менее 10% от общего. Для этих образцов были проведены дополнительные исследования ТТГ, общих и свободных фракций Т3 и Т4. В результате все показатели укладывались в пределы нормальных значений, что свидетельствует об отсутствии влияния на функцию ЩЖ снижения содержания I в липофильной фракции.

**Figure:**
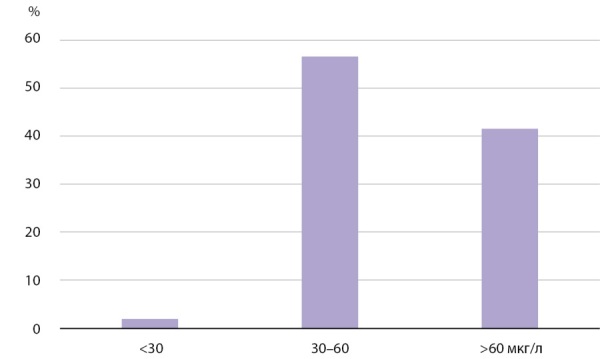
Рисунок 1. Частотное распределение концентрации I в сыворотке крови.

**Figure:**
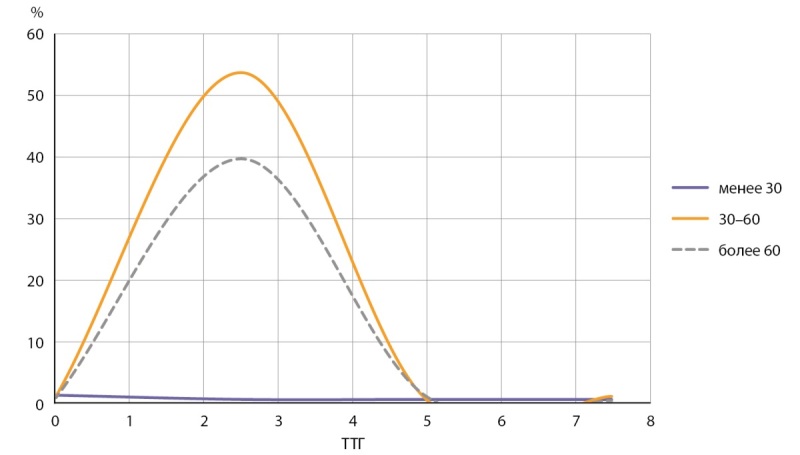
Рисунок 2. Показатели ТТГ в группах с уровнем I в сыворотке крови менее 30 мкг/л, 30–60 мкг/л и более 60 мкг/л.

**Table:** Таблица 1. Медианная концентрация микроэлементов в сыворотке крови (мкг/л) *Референсные значения изучаемых показателей установлены лабораторией метаболомных исследований ГНЦ РФ ФГБУ «НМИЦ эндокринологии» Минздрава России. Не имеют статистически значимых возрастных и половых различий.**В данном столбце указано среднее значение. Стандартное отклонение для йода — 17,7; для селена — 22,6; для цинка — 529,7.Уровень статистической значимости результатов менее/равен 0,02 (р≤0,02).

Показатель	Референсн. значения*	Респ. Крым	Респ. Тыва	Брянск. обл.	Тульск. обл.	Чеченск. Респ.	Среднее значение**
I	30–60	40	37,9	43,4	84,3	63,4	60,68
Se	40–180	68,8	67,8	62,1	96,3	121,9	83,38
Zn	1000–2000	1693,6	1712,8	517,2	754,6	626,9	632,9

Проведен сравнительный анализ результатов определения I в моче церий-арсенитным методом и методом ИСП-МС (таблица 2).

**Table:** Таблица 2. Медианная концентрация йода в сыворотке крови и моче, мкг/л * Для метода ИСП-MС референсные значения 100–200 мкг/л установлены лабораторией метаболомных исследований ГНЦ РФ ФГБУ «НМИЦ эндокринологии» Минздрава России.** Представлены результаты обследования детей допубертатного возраста (8–10 лет) (Трошина Е.А., Маколина Н.П., Сенюшкина Е.С., Никанкина Л.В., Малышева Н.М., Фетисова А.В. Йододефицитные заболевания: текущее состояние проблемы в Брянской области // Проблемы эндокринологии. — 2021. — Т. 67. — №4. — С. 84-93. doi: https://doi.org/10.14341/probl12793; Трошина Е.А., Сенюшкина Е.С., Маколина Н.П., Абдулхабирова Ф.М., Никанкина Л.В., Малышева Н.М., Репинская И.Н., Дивинская В.А. Йододефицитные заболевания: текущее состояние проблемы в Республике Крым // Клиническая и экспериментальная тиреоидология. — 2020. — Т. 16. — №4. — С. 19-27. doi: https://doi.org/10.14341/ket12700; Трошина Е.А., Мазурина Н.В., Сенюшкина Е.С., Маколина Н.П., Галиева М.О., Никанкина Л.В., Малышева Н.М., Даржаа А.Б., Сенги Ю.С. Мониторинг эффективности программы профилактики заболеваний, связанных с дефицитом йода, в Республике Тыва // Проблемы эндокринологии. — 2021. — Т. 67. — №1. — С. 60-68. doi: https://doi.org/10.14341/probl12715).Уровень статистической значимости результатов менее/равен 0,02 (р≤0,02).

Метод исследования/исследуемый материал	Брянская обл.	Респ. Крым	Респ. Тыва
ИСП-MС, сыворотка крови	43,4	39,75	38,8
Церий-арсенитный метод, моча**	98,3	97	153
ИСП-MС, моча*	108,2	124,3	107,6

Результаты исследования концентрации I в моче методом ИСП-MС свидетельствуют о низконормальных значениях в общей сплошной выборке по регионам. Нами подтверждено, что церий-арсенитный метод исследования I в моче в целом сопоставим с методом ИСП-МС.

Медиана концентрации Se в сыворотке крови составила 83,38 мкг/л (таблица 1), что соответствует референсным значениям. Уровень Se в сыворотке крови менее 40 мкг/л выявлен у 2,2% обследуемых. Из 1150 человек сформировано две группы: 1) с уровнем Se в сыворотке крови менее 100 мкг/л; 2) с уровнем Se в сыворотке крови более 100 мкг/л (таблица 3). При сравнении сформированных групп выяснилось, что в группе с низконормальными показателями Se частота встречаемости аутоиммунной патологии на 5% выше, чем в группе сравнения. Полученные данные подтверждают важнейшую биологическую роль Se, связанную с антиоксидантной защитой. Не исключено, что посредством антиоксидантного воздействия возможно предотвращение воспалительной активности при АИЗ ЩЖ.

**Table:** Таблица 3. Частота встречаемости АИЗ ЩЖ в зависимости от уровня Se в сыворотке крови *Медианная концентрация селена в сыворотке крови. Полученные значения не имеют статистически значимых возрастных и половых различий. Уровень статистической значимости результатов менее/равен 0,02 (р≤0,02).

Уровень Se в сыворотке крови (мкг/л)*	Частота встречаемости АИЗ ЩЖ (ХАИТ, болезнь Грейвса), %
менее 100 (n=205)	22%
более 100 (n=205)	17 %

В нашем исследовании 60,3% взрослого населения имели уровень Zn менее 1000 мкг/л (Брянская и Тульская обл., Чеченская Респ.) (таблица 1, рис. 3). Медианная концентрация Zn в сыворотке крови составила 632,9 мкг/л (таблица 1). Полученные результаты могут быть обусловлены особенностями питания населения регионов, содержанием микроэлементов в почве и воде по каждому региону (например, в Брянской обл., по данным исследования V.Yu. Berezkin и соавт., содержание йода в поверхностных водах низкое и составляет 6,76 мкг/л [20]). Важно отметить, что некоторые тяжелые металлы потенциально могут влиять на метаболизм Zn.

**Figure:**
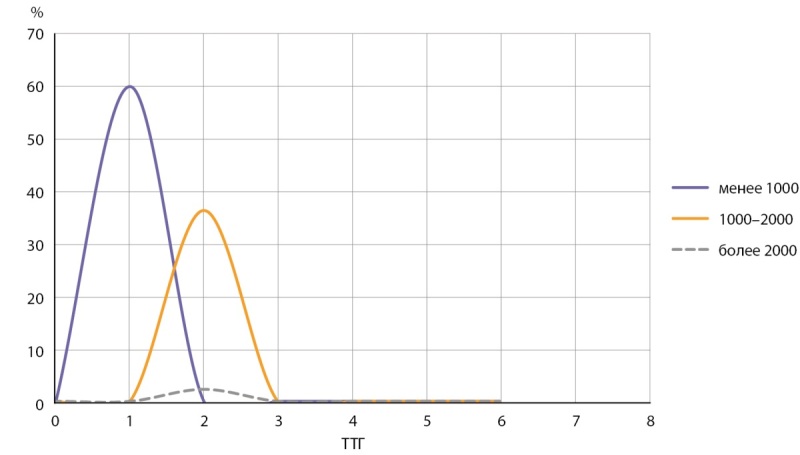
Рисунок 3. Показатели ТТГ в группах с уровнем Zn в сыворотке крови менее 1000 мкг/л, 1000–2000 мкг/л и более 2000 мкг/л.

Не выявлено взаимосвязи между содержанием в сыворотке крови Zn и ZnT8A, ЩФ и SOD1, в т.ч. в сопоставлении полученных данных у носителей АТ-ТПО и в группе сравнения (таблица 4).

**Table:** Таблица 4. Показатели Zn и дополнительные диагностические маркеры в сопоставлении со структурно-функциональными характеристиками ЩЖ у носителей АТ-ТПО и в группе сравнения Полученные значения не имеют статистически значимых возрастных и половых различий. Уровень статистической значимости результатов менее/равен 0,02 (р≤0,02).

Исследуемая группа	Zn (мкг/л)	SOD1 (нг/мл)	ЩФ (Ед/л)	ZnT8А	ТТГ↑	ТТГ↓	Узл. зоб	ХАИТ	Без стр. патологии
АТ-ТПО”+”(n=95)	644,4	117,2	70,3	249,8	9,2%	9,4%	10,5%	75%	14,5%
АТ-ТПО”-”(n=95)	744,6	102,4	66,1	242	N	N	N	N	100%

В таблице 5 представлена распространенность и структурная патология ЩЖ по регионам.

**Table:** Таблица 5. Распространенность ультразвуковых признаков узлового/многоузлового зоба и аутоиммунных заболеваний ЩЖ Полученные значения не имеют статистически значимых возрастных и половых различий. Уровень статистической значимости результатов менее/равен 0,02 (р≤0,02).

Структурная патология ЩЖ (поvданным УЗИ)	Крым (n=253)	Тыва (n=204)	Брянск. обл. (n=58)	Тульская обл. (n=308)	Чеченская Респ. (n=329)
узловой/многоузловой зоб (%)	13,3	40,6	56	27,3	24
ХАИТ, ДТЗ (%)	11,3	10,4	24	15,4	25

## ОБСУЖДЕНИЕ

Целью проведенного исследования было изучение роли эссенциальных микроэлементов в патогенезе ЙДЗ и АИЗ ЩЖ и обоснование выбора биомаркеров обеспеченности и аналитических методов определения. Впервые в России проведено когортное обследование взрослого населения, проживающего в регионах с доказанным хроническим дефицитом йода тяжелой и легкой степени тяжести, но различающихся по экологическим характеристикам и обеспеченности другими микронутриентами (предшествующее радиационное загрязнение, обеспеченность Se и Zn). Впервые для элементного анализа нами применен метод ИСП-МС и проведено его сопоставление по чувствительности, специфичности с церий-арсенитным методом.

Результаты исследования позволяют подтвердить взаимосвязь между коморбидностью ЙДЗ и АИЗ ЩЖ и микронутриентными дефицитами.

Медианная концентрация I сыворотки крови отличается по регионам. Метод ИСП-МС и церий-арсенитный метод в целом сопоставимы и доказали дефицит I легкой степени тяжести во всех обследованных регионах. Однако метод ИСП-МС требует дальнейшего совершенствования ввиду технических особенностей и ограничения по объему выборки изучаемой популяции. При оценке содержания I в липофильной фракции (n=57) не обнаружено влияния снижения содержания I на функцию ЩЖ. Следует отметить, что окончательный вывод можно получить только на более широкой когорте пациентов.

В нашем исследовании не выявлено дефицита Se ни в одном из обследуемых регионов. Однако при сравнении групп с концентрацией Se в сыворотке крови менее 100 мкг/л и более 100 мкг/л в группе с низконормальной концентрацией Se частота встречаемости АИЗ ЩЖ на 5% больше. Полученные результаты позволяют подтвердить важнейшую биологическую роль селена как антиоксиданта. Селен входит в состав селенсодержащих белков, локализующихся в больших количествах в ЩЖ. Нарушения биологической функции селенсодержащих белков играют важную роль в развитии аутоиммунных заболеваний ЩЖ, а дефицит селена инициирует и способствует их прогрессированию.

Результаты проведенного исследования свидетельствуют о дефиците Zn у 60,3% взрослого населения. Не выявлено взаимосвязи между содержанием в сыворотке крови Zn и дополнительных маркеров обеспеченности: ZnT8A, ЩФ, SOD1, в т.ч. в сопоставлении полученных данных у носителей АТ-ТПО и в группе сравнения. Таким образом, оценивать дополнительные специфические индикаторы обеспеченности цинком не является убедительным. Тем не менее исследование данных маркеров требует более детального и углубленного изучения. Следует отметить, что в регионах с дефицитом Zn частота встречаемости АИЗ ЩЖ и узлового/многоузлового зоба в среднем на 10% выше, чем в регионах с оптимальной обеспеченностью Zn. Следовательно, обеспеченность населения Zn наряду с обеспеченностью I и другими микроэлементами напрямую связана с тиреоидной патологией.

Важно отметить, что проведение любого исследования во многом зависит от точности результатов лабораторных исследований, а также от референсных интервалов, позволяющих правильно интерпретировать полученные данные. В нашем исследовании референсные значения изучаемых показателей установлены лабораторией метаболомных исследований ГНЦ РФ ФГБУ «НМИЦ эндокринологии» Минздрава России.

Диапазон референсных значений варьирует в зависимости от исследуемой популяции пациентов и используемых тестовых систем. Различия между лабораториями могут возникать из-за особенностей используемого оборудования, химических реагентов и методик анализа. Следовательно, для большинства лабораторных исследований не существует универсально применимого эталонного значения.

## Сопоставление с другими публикациями

Сравнивая полученные нами результаты с другими подобными исследованиями, необходимо отметить, что в странах Европы, США, Китае и др. на протяжении многих лет изучается взаимосвязь дефицита микроэлементов и заболеваний ЩЖ [2][21–26]. Наряду с дефицитом других микроэлементов, низкие уровни Zn и Se повышают риск развития узлового зоба, увеличивают выработку антител к ткани ЩЖ, инициируют и способствуют прогрессированию ХАИТ и болезни Грейвса. Многие исследования демонстрируют преимущества ежедневного поступления с пищей Se и Zn в лечении АИЗ ЩЖ и узлового зоба [2][21–26]. В большинстве рекомендаций относительно ежедневного перорального приема микроэлементов взрослыми старше 18 лет Se составляет от 50 до 70 мкг/день, Zn — 8–15 мг/день.

## Клиническая значимость результатов

Клиническая значимость полученных нами результатов состоит в разработке методики определения эссенциальных микроэлементов в сыворотке крови и моче как для эпидемиологических, так и для клинических исследований; уточнении лабораторных критериев для выявления ассоциированных с патологией щитовидной железы влияний дефицитов эссенциальных микроэлементов. В дальнейшем предложенные методы и диагностические критерии будут включены в соответствующие клинические рекомендации и программы по профилактике и мониторингу заболеваний щитовидной железы.

## Ограничения исследования

Ограничением нашего исследования является недостаточно широкий охват населения регионов РФ. Тем не менее, по нашему мнению, выборка достаточно репрезентативна с учетом предшествующих обследований. Не проводилось определение содержания GPX1, как дополнительного маркера дефицита Se. Нами не учитывалось возможное влияние ряда других эссенциальных микроэлементов на полученные результаты.

## Направления дальнейших исследований

В дальнейшем планируется расширение выборки для липофильной фракции, ZnT8A.

## ЗАКЛЮЧЕНИЕ

Исследование обеспеченности эссенциальными микроэлементами населения регионов РФ, имеющих высокую распространенность заболеваний щитовидной железы, доказало взаимосвязь между содержанием и метаболизмом I, Se, Zn и риском развития йододефицитных и аутоиммунных заболеваний щитовидной железы. Более половины взрослого населения регионов РФ — 60,3% — имеют дефицит Zn. Частота встречаемости аутоиммунных заболеваний ЩЖ и узлового/многоузлового зоба у данной части населения выше на 10%. В группе с низконормальными показателями Se частота встречаемости аутоиммунной патологии щитовидной железы также выше на 5%, чем в группе сравнения. Используемый нами метод ИСП-МС позволил предложить собственные референсные значения I, Se, Zn. При исследовании йода в моче метод ИСП-МС сопоставим с церий-арсенитным методом по чувствительности и специфичности, но требует дальнейшего совершенствования ввиду технических особенностей и ограничения по объему выборки изучаемой популяции. Определение дополнительных маркеров дефицита цинка (ZnT8A, ЩФ, SOD1) не дало убедительных доказательств целесообразности их определения в рутинной практике.

Несмотря на большое количество проведенных, подобно нашему, исследований, как в России, так и в зарубежных странах и широкое понимание важности эссенциальных микроэлементов в питании населения, конкретные знания среди клиницистов остаются ограниченными. Для большинства микроэлементов нет четкого понятия «дефицит», «недостаточность», не разработаны методики определения и системы мониторинга, отсутствуют меры профилактики и методы лечения заболеваний, связанных с микронутриентными дефицитами. Таким образом, необходима разработка новых и дополнение существующих клинических рекомендаций и программ по профилактике, мониторингу и лечению заболеваний щитовидной железы с акцентом на ключевые эссенциальные микроэлементы.

## ДОПОЛНИТЕЛЬНАЯ ИНФОРМАЦИЯ

Источники финансирования. Исследование проведено при финансировании РНФ (проект №22-15-00135 «Научное обоснование, разработка и внедрение новых технологий диагностики коморбидных йододефицитных и аутоиммунных заболеваний щитовидной железы, в том числе с использованием возможностей искусственного интеллекта»).

Конфликт интересов. Авторы декларируют отсутствие явных и потенциальных конфликтов интересов, связанных с публикацией настоящей статьи.

Участие авторов. Концепция и дизайн исследования — Трошина Е.А., Платонова Н.М., Иоутси В.А., Смолин Е.С., Никанкина Л.В., Зураева З.Т.; сбор и обработка материала, статистическая обработка, написание текста — Сенюшкина Е.С.; редактирование, утверждение окончательного варианта статьи, ответственность за целостность всех частей статьи — все авторы. Все авторы одобрили финальную версию статьи перед публикацией, выразили согласие нести ответственность за все аспекты работы, подразумевающую надлежащее изучение и решение вопросов, связанных с точностью или добросовестностью любой части работы.
